# Screening for Influenza A(H1N1)pdm09, Auckland International Airport, New Zealand

**DOI:** 10.3201/eid1805.111080

**Published:** 2012-05

**Authors:** Michael J. Hale, Richard S. Hoskins, Michael G. Baker

**Affiliations:** Auckland District Health Board, Auckland, New Zealand (M.J. Hale, R.S. Hoskins);; University of Otago, Wellington, New Zealand (M.G. Baker)

**Keywords:** pandemic, communicable diseases, influenza, viruses, emigration and immigration, mass screening, sensitivity, program evaluation, airport, influenza, New Zealand, influenza A(H1N1)pdm09, pandemic (H1N1) 2009, H1N1, pH1N1

## Abstract

Entry screening for influenza A(H1N1)pdm09 at Auckland International Airport, New Zealand, detected 4 cases, which were later confirmed, among 456,518 passengers arriving April 27–June 22, 2009. On the basis of national influenza surveillance data, which suggest that ≈69 infected travelers passed through the airport, sensitivity for screening was only 5.8%.

The virus that caused the 2009 influenza pandemic, hereafter referred to as influenza A(H1N1)pdm09, is mainly spread internationally by air travel (*1*). To prevent or delay such spread, during the pandemic many countries initiated screening of air travelers arriving at airports, even though these measures have not been recommended by the World Health Organization (*2*). On April 25, 2009, New Zealand was one of the first countries outside the Americas to confirm influenza A(H1N1)pdm09 in arriving airline passengers (*3*). During April 27–June 22, 2009, at the direction of the Ministry of Health, the Auckland Regional Public Health Service began a screening program at Auckland International Airport.

Protocols for border screening were updated throughout the pandemic and evolved as new information became available. Screening was initially applied to all passengers arriving or transferring on flights from countries where community transmission of influenza A(H1N1)pdm09 virus was occurring. The screening program included the following (Figure):

**Figure:**
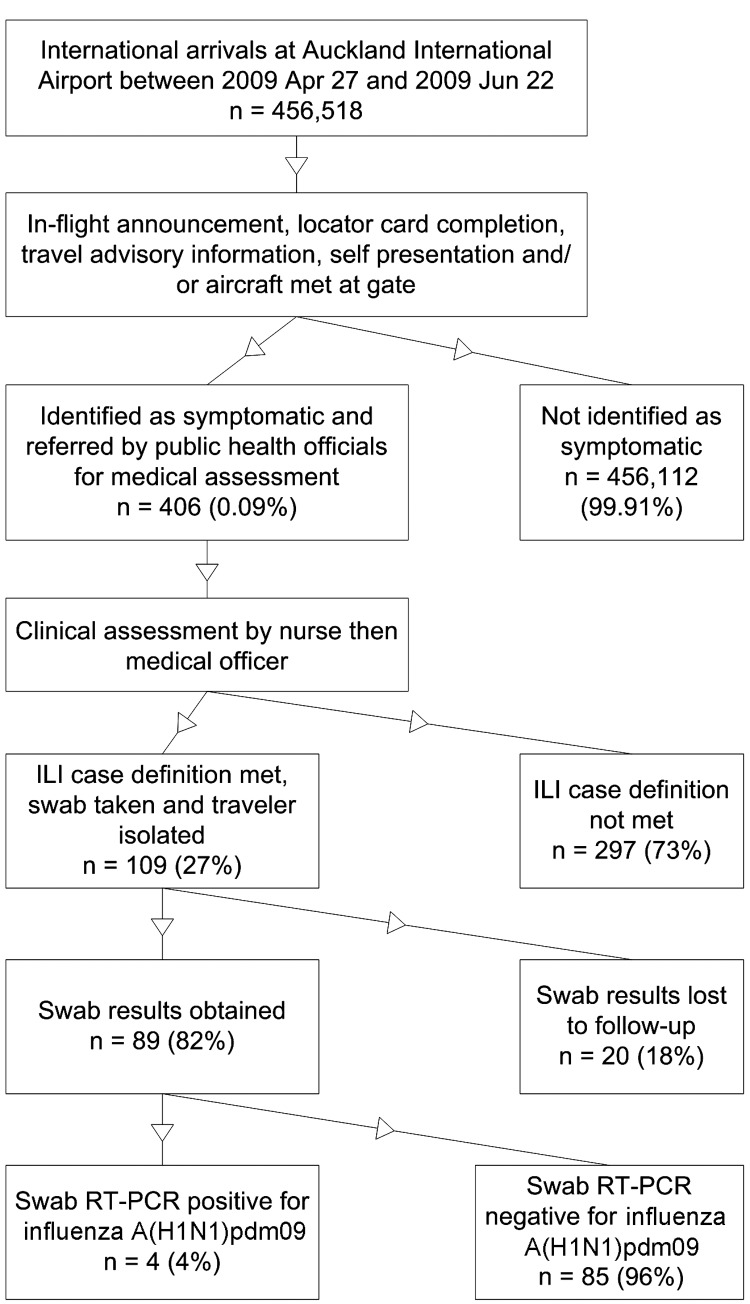
Process used to screen arriving international passengers for influenza A(H1N1)pdm09, Auckland International Airport, New Zealand, April 27–June 22, 2009. ILI, influenza-like illness; RT-PCR, reverse transcription PCR.

- All flights notified New Zealand of the health of passengers and crew on board before landing; if indicated, the aircraft was met by public health officials who triaged these travelers.
- Cabin crew announced a scripted health message requesting passengers to identify themselves if symptomatic; after disembarkation, all passengers passed through a public health checkpoint where signage encouraged ill travelers to seek screening.
- Information about influenza A(H1N1)pdm09 was available, those with symptoms could self-declare, and public health officials visually inspected arriving passengers and approached those with apparent symptoms.
- Neither thermal scanning nor active screening of every arriving passenger was used.

Each unwell passenger and crew member was screened for influenza-like illness by a nurse and assessed by a medical officer if illness met the definition of a suspected case. Those whose illness met the case definition had nasopharyngeal swabs taken, were offered oseltamivir, and were sent home or to a facility for isolation. Reverse transcription PCR (RT-PCR) was used to confirm infection. Screening was escalated on April 29 to include all passengers arriving from other countries and stopped on June 22.

## The Study

We quantified the results of entry screening for influenza A(H1N1)pdm09 at Auckland International Airport. Using the information generated during screening, we retrospectively estimated the number of infected travelers who actually passed through the airport. To estimate the sensitivity of screening, we then compared screening findings with the expected number of infected travelers who passed through the airport. Ethical approval was received from the Northern X Regional Ethics Committee of the New Zealand Ministry of Health.

The number of screened passengers was obtained from airport records. The numbers of crew members on inbound international aircraft were estimated by using averages for flights into Auckland. The number of travelers detected at each step and referred to the next step of the screening process was obtained from Auckland Regional Public Health Service records. Virologic test results were extracted from laboratory information systems. A confirmed case was one that met the current case definition (as published on the Ministry of Health website, www.health.govt.nz) and one for which RT-PCR result was positive.

We estimated the number of infected travelers screened as the total number of confirmed cases in New Zealand during this period, multiplied by the proportion of overseas-acquired cases, and the proportion of international travelers arriving at the airport. On April 30, 2009, nonseasonal influenza A (H1N1) was made notifiable, and these data were collated on the national surveillance database (EpiSurv) (www.surv.esr.cri.nz/episurv). The proportion of infected travelers who acquired the infection overseas was extrapolated from Ministry of Health records of the first 100 cases of pandemic (H1N1) 2009 because this information was not collected for all travelers with confirmed infection. The proportion of travelers who passed through the airport was determined from Statistics New Zealand (www.stats.govt.nz) arrivals records. Confidence intervals were calculated by using the online calculator for screening on Open Epi (*4*).

During the screening period, 456,518 international travelers were screened; 406 (0.09%) of these were referred for medical assessment. Of those, 109 (27%) met the case definition and received virologic testing. RT-PCR results were located for 89 (82%), among which 4 were positive. A total of 312 cases were confirmed. The proportion of travelers who acquired the infection overseas was 32%. The proportion who passed through the airport was 69%. The expected number of infected travelers estimated to have passed through the border during the screening program was therefore 69, giving an estimated sensitivity of 5.8% (95% CI 2.3%–14.0%).

## Conclusions

The influenza A(H1N1)pdm09 screening program at Auckland International Airport had low sensitivity. This form of border screening is therefore unlikely to have substantially delayed spread of the pandemic into New Zealand in 2009.

Limitations of influenza screening include the high proportion of asymptomatic infected travelers (*5*), incubation of infections acquired before or during a flight (*3*), reliance on self-identification, limitations of case definitions, and limitations of thermal scanning (*6*). Modeling data have shown that the ability of border screening to delay global pandemic influenza is closely linked to the effectiveness of the screening process or travel restriction used. To delay influenza spread by 1.5 weeks, border restrictions need to reduce imported infections by 90% (*7*). The entry screening program we describe does not meet these standards.

The potential effectiveness of screening arriving travelers to prevent or delay influenza epidemics has been debated. Mathematical models and literature reviews have argued for (*7**,**8*) and against (*9**–**11*) this approach. Some authors have found that entry screening for respiratory conditions or influenza A(H1N1)pdm09 is insensitive and not cost-effective (*12*). Border screening did not substantially delay local transmission of influenza A(H1N1)pdm09 (*13*).

This study has several limitations, particularly with regard to estimating the number of infected travelers who would have passed through the airport during the screening period. Most cases of illness acquired overseas would probably not have been notified, particularly those in patients with mild illness who did not see a doctor or who saw a doctor but did not receive a diagnosis. The estimated proportion of overseas-acquired cases was based on data from the first 100 cases and would have decreased as the pandemic progressed. The net effect of these factors is unknown, but they would probably have increased the estimated number of undetected infected travelers passing through screening, thereby further reducing the estimated sensitivity of screening.

Border screening might be conducted for reasons other than preventing or delaying an epidemic. It might provide public assurance and confidence that something is being done (*14*). The communication of health information and advice on how to seek treatment is consistently recommended as a pandemic prevention strategy (*12**,**15*) and is usually delivered as part of border screening programs. These benefits need to be balanced against the considerable resources used, opportunity cost (resources used for this activity and thereby unavailable for other activities), uncertain effectiveness, and inconvenience of border screening.

To delay or prevent influenza entry at borders, influenza screening needs to be considerably more effective than the mostly passive program described here. We hope that during this interepidemic period, a major international review of the role of international air travel in the dissemination of emerging infectious diseases will be conducted to identify effective interventions. Such a review should consider systemwide approaches, including exit screening, standardized health declarations, active screening of individual passengers (including use of rapid laboratory tests and thermal scanning), passenger tracking, policies and practices that support sick travelers wishing to defer travel, and circumstances where airline travel should be suspended entirely.
